# Metastasis of Hepatocellular Carcinoma to the Urinary Bladder: A Case Report and Literature Review

**DOI:** 10.1002/iju5.70189

**Published:** 2026-05-03

**Authors:** Eri Fukagawa, Suguru Oka, Kazushige Sakaguchi, Shigeki Yamamoto, Masako Ikemura, Norio Akuta, Shinji Urakami

**Affiliations:** ^1^ Department of Urology Toranomon Hospital Tokyo Japan; ^2^ Department of Hepatology Toranomon Hospital Tokyo Japan; ^3^ Department of Pathology Toranomon Hospital Tokyo Japan

**Keywords:** hepatocellular carcinoma, neoplasm metastasis, transurethral resection of bladder, urinary bladder, urinary bladder neoplasms

## Abstract

**Introduction:**

Although secondary bladder tumors from distant metastases are rare, those originating from hepatocellular carcinoma are particularly uncommon, with only a few cases reported to date.

**Case Presentation:**

An 81 year old male with hepatocellular carcinoma developed a rapidly growing 23 mm‐sized bladder tumor during systemic therapy with atezolizumab and bevacizumab. Cystoscopy revealed a pedunculated, nonpapillary tumor on the posterior wall of the bladder, and transurethral resection was urgently performed due to bleeding. Histopathology and immunohistochemistry confirmed bladder metastasis of hepatocellular carcinoma.

**Conclusion:**

Although rare, rapidly enlarging bladder tumors in patients with hepatocellular carcinoma should be considered as possible metastatic lesions in the differential diagnosis.

## Keynote Message

- Although hepatocellular carcinoma is associated with poor prognosis due to a high risk of recurrence and distant metastasis, bladder metastasis remains extremely rare and has been reported only in isolated cases.
- This report presents a rare case of secondary bladder tumor originating from hepatocellular carcinoma.
- Secondary lesions should be considered as a differential diagnosis of bladder tumors in patients with a history of hepatocellular carcinoma.

**Abbreviations**
ATZ/BEV: Atezolizumab and Bevacizumab
BSC: best supportive care
Cx: chemotherapy
HCC: hepatocellular carcinoma
LVB: Lenvatinib
N/R: not reported
Rx: radiotherapy
TACE: transcatheter arterial chemoembolization
TUR‐Bt: transurethral resection of bladder tumor

## 1 Introduction

Although secondary bladder tumors from distant metastases are rare, those originating from hepatocellular carcinoma (HCC) are particularly uncommon, with only a few cases reported to date. This report presents a rare case of urinary bladder metastasis from HCC with a review of the literature.

## 2 Case Presentation

**FIGURE 1:**
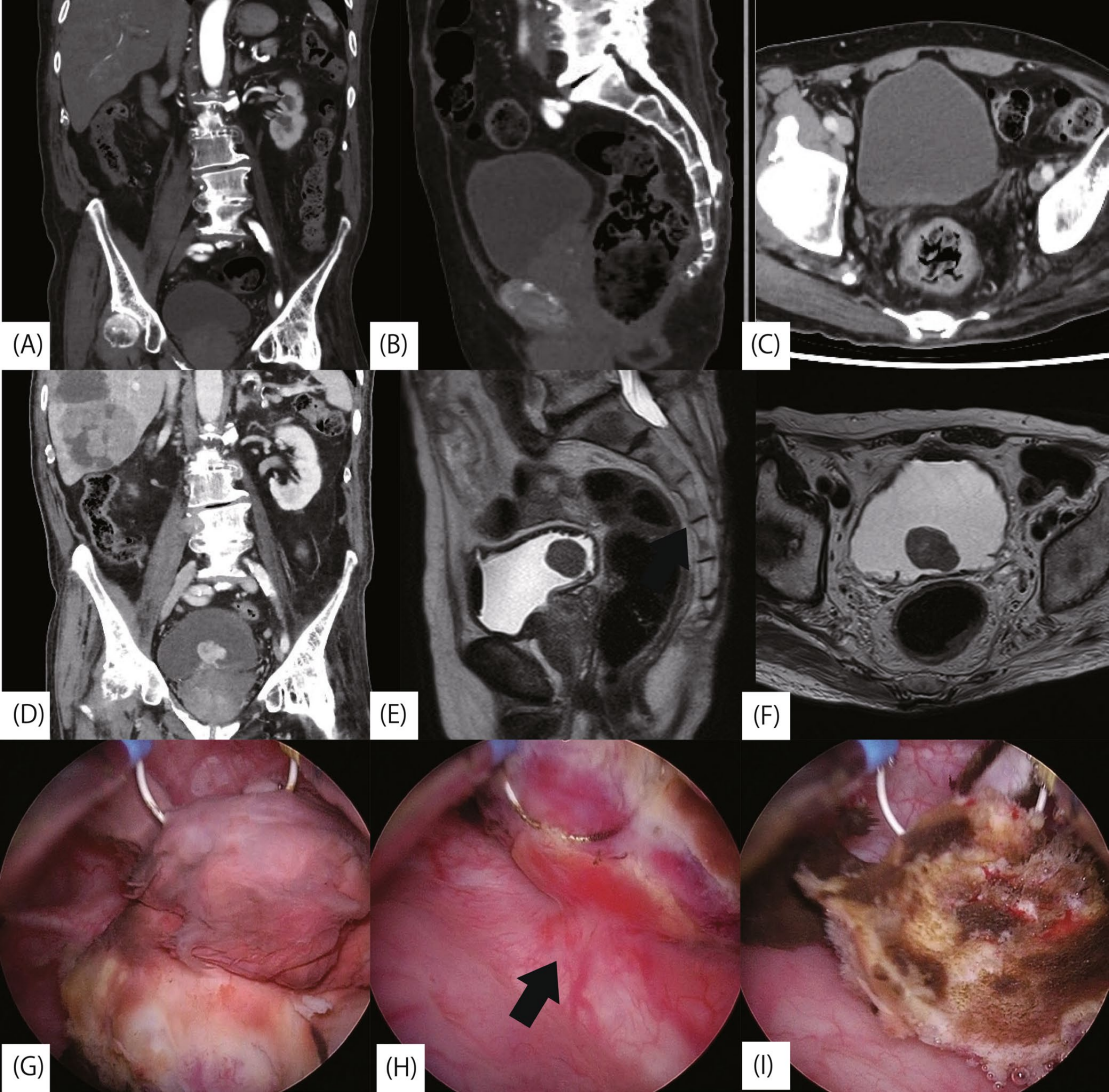
CT images obtained 3 months earlier showed no evidence of a bladder tumor (A–C). At diagnosis, CT revealed a 23 mm tumor on the posterior wall of the bladder (D), and MRI suggested no evidence of obvious muscle invasion (E, F). Intraoperative findings showed a nonpapillary tumor located on the posterior wall of the bladder (G), characterized by a slender pedunculated base (H; indicated by arrow) and a necrotic center (I).

An 81‐year‐old male with a history of transfusion‐related hepatitis C virus infection and chronic hepatitis had achieved a sustained virological response 9 years earlier following combination therapy with ledipasvir and sofosbuvir. However, he subsequently developed HCC in segment 4 of the liver, and he underwent laparoscopic hepatectomy 1 year ago. Four months prior, he developed lower limb neuropathy which revealed multiple bone metastases including the vertebrae, and he underwent laminectomy and radiotherapy. One month prior, transcatheter arterial chemoembolization (TACE) was performed for multinodular recurrent lesions. Due to increasing tumor marker levels postoperatively, systemic therapy with atezolizumab and bevacizumab (ATZ/BEV) was initiated the following month, with performance status 2 and Child‐Pugh class A. At that time, a computed tomography performed prior to treatment initiation incidentally revealed multiple pulmonary nodules as well as a 23‐mm bladder tumor (Figure 1D), neither of which had been observed 3 months earlier (Figure 1A–C), prompting referral to our department.

Cystoscopy demonstrated a pedunculated, nonpapillary tumor on the posterior wall of the bladder. Urine cytology was classified as Class I. Magnetic resonance imaging suggested a tumor arising from the bladder wall without evidence of obvious muscle invasion (Figure 1E,F). As bevacizumab is associated with an increased risk of bleeding‐related complications [1], a temporary discontinuation of bevacizumab was considered necessary to safely perform surgical resection. However, interruption of treatment risked compromising HCC disease control, leading to a clinical dilemma regarding treatment prioritization.

Three weeks after the examination, while hospitalized for the next scheduled ATZ/BEV cycle, the patient suddenly developed gross hematuria that had not been previously observed. He subsequently developed clot retention, necessitating continuous bladder irrigation and blood transfusion. Since active bleeding precluded further chemotherapy, an emergent transurethral resection of the bladder tumor (TUR‐Bt) was performed. Intraoperatively, a single, slender‐pedunculated, nonpapillary tumor with a necrotic center was identified on the posterior bladder wall (Figure 1G–I).

**FIGURE 2:**
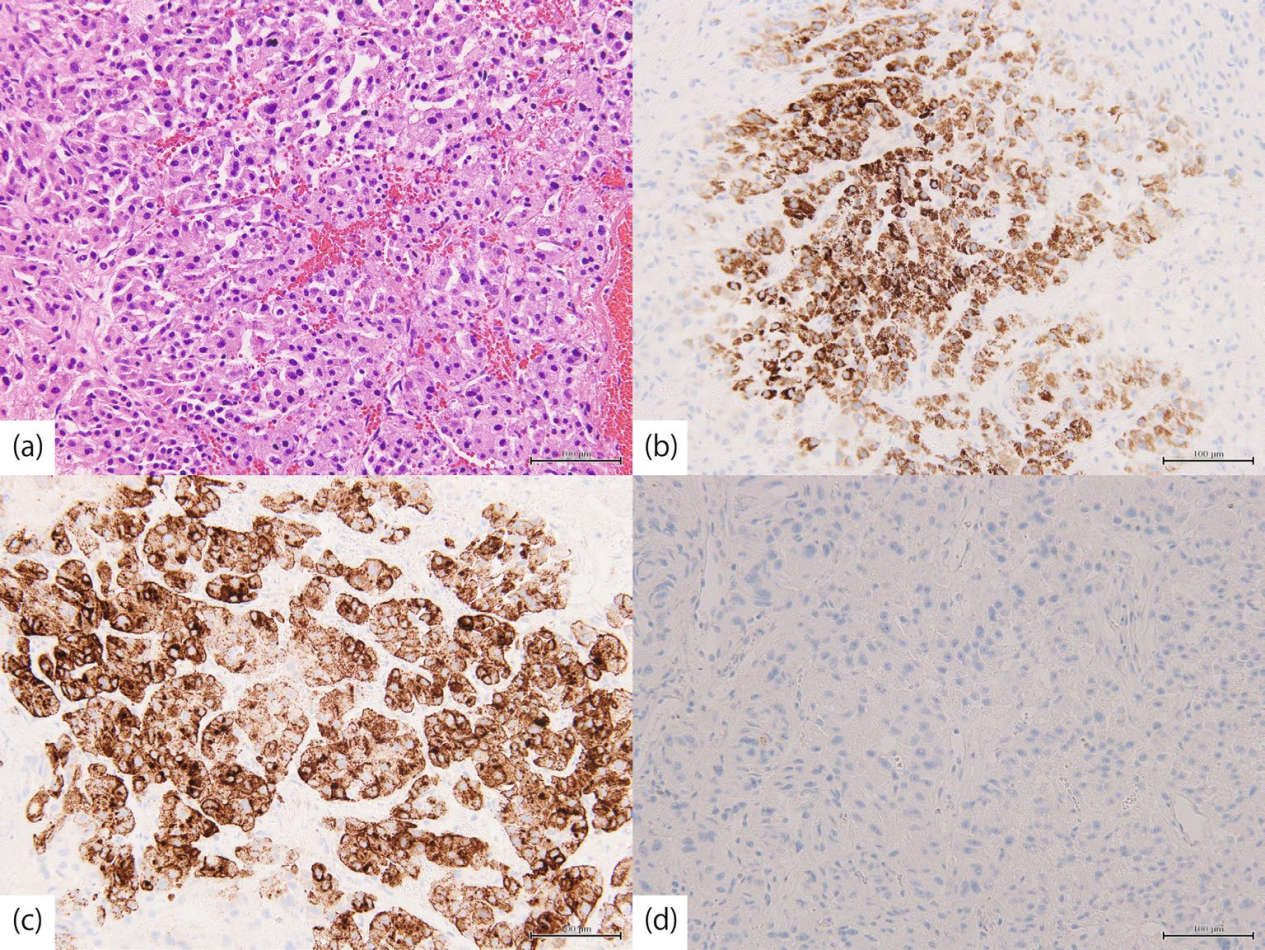
Histopathological findings of specimens (scale bar: 100 μm). (a) Hematoxylin and eosin staining. Immunohistochemical examinations demonstrating positive staining for (b) hepatocyte and (c) Glypican‐3, and negative staining for (d) GATA‐binding protein 3.

Histologically, the tumor was composed of cells with chromatin‐rich, enlarged nuclei and pale eosinophilic cytoplasm, and exhibited infiltrative growth in a nested pattern, with areas showing formation of cord‐like structures. Immunohistochemical staining for Hepatocyte and Glypican‐3 was positive, whereas staining for GATA‐binding protein 3 was negative (Figure 2). These findings confirmed the lesion to be the bladder metastasis of HCC. Histopathological evaluation of the depth of invasion was limited because the tumor showed extensive necrotic changes, and the muscularis propria could not be identified in the resected specimen.

The postoperative course was uneventful, and the patient was discharged on postoperative day 4. However, his general condition deteriorated due to progression of the primary disease, and he died 25 days after surgery.

## 3 Discussion

HCC ranks as the sixth most prevalent form of cancer and is the third leading cause of cancer‐related mortality worldwide [2]. HCC typically arises in the setting of chronic liver inflammation leading to cirrhosis, most commonly caused by hepatitis B and C virus infections, alcoholic liver disease, and metabolic dysfunction‐associated steatotic liver disease. Due to this underlying liver damage, HCC is associated with a high recurrence rate even after curative treatment, and more than half of patients reportedly experience intrahepatic recurrence within 2 years of diagnosis [3]. Although the prognosis has improved over time, the 23rd Nationwide Follow‐up Survey of Primary Liver Cancer in Japan reported a median overall survival of 67.2 months among 94 289 registered patients with HCC, with 5‐ and 10‐year survival rates of 53.6% and 27.8%, respectively [3]. Distant metastases from HCC most frequently involve the lungs (31.7%), followed by lymph nodes (29.9%), bones (21.2%), peritoneum (6.4%), adrenal glands (6.3%), brain (0.6%), and other sites (4.0%) [3].

In contrast, secondary bladder tumors account for only 2%–3% of all bladder neoplasms. Most represent direct invasion from tumors arising in adjacent organs such as the genitourinary tract and lower gastrointestinal tract. In cases of secondary bladder tumors originating from distant primary malignancies, hematogenous spread is considered the most plausible mechanism [4]. In the present case, there was no evidence of direct invasion from adjacent organs, and given the presence of multiple systemic metastases, hematogenous dissemination is likely to have played a central role in the development of the bladder lesion. Distant metastases are rare and most commonly arise from the stomach (4.3% of secondary bladder tumors), skin (3.9%), lung (2.8%), and breast (2.5%) [5].

Bladder metastasis from HCC is exceedingly rare; a PubMed search for case reports with full‐text available identified only eight cases to date, including ours, as shown in Table 1 [6, 7, 8, 9, 10, 11, 12]. Five out of the eight cases originated from Asia, which is consistent with a previous report indicating that, in 2020, Asian countries accounted for 72.5% of the global incidence and 73.3% of global mortality from HCC [13]. Although previous reports have provided limited information on the prognosis of bladder metastasis from HCC, according to a study by El‐Taji et al., the median survival for patients from diagnosis of a secondary bladder tumor was 272 days. Among cases attributable to true distant metastases, specifically those originating from breast cancer, lymphoma, renal cell carcinoma, and gastric cancer, the median survival was even shorter, at 201 days [14]. As observed in our case, previous reports have frequently described bladder lesions using terms such as “pedunculated,” “polypoid,” and “protruding” [6, 9, 10, 11, 12], suggesting that secondary bladder tumors originating from HCC often mimic the appearance of primary bladder tumors both radiologically and endoscopically. Clinicians should remain vigilant for atypical metastatic presentations of HCC and should consider histological confirmation when bladder lesions are identified in patients with a known history of primary HCC. Prompt and accurate diagnosis is crucial to guide treatment strategy and minimize therapeutic delays.

**TABLE 1:** Characteristics of reported cases of urinary bladder metastases originating from hepatocellular carcinoma.

No.	Author	Year	Location	Age	Sex	HCC etiology	Previous treatment	Other sites of metastasis	Diagnosis	Sites of bladder tumor	Treatment after diagnosis	Prognosis
1	Franks et al. [6]	1999	United States	51	Male	Hepatitis C alcoholic	Liver transplantation	Lung	TUR‐Bt	Dome	Cx; leucovorin and 5‐fluorouracil	N/R
2	Al‐Brahim et al. [7]	2004	Canada	83	Male	N/R	None	Adrenal gland	Biopsy	Right lateral	N/R	N/R
3	Chung et al. [8]	2007	Taiwan	58	Female	Hepatitis C	None	Unknown	TUR‐Bt	Posterior	BSC	Died of disease 5 months after TUR‐Bt
4	Yasutomi et al. [9]	2020	Japan	89	Male	Alcoholic	TACE	None	TUR‐Bt	Anterior	N/R	N/R
5	Kim et al. [10]	2022	Korea	60	Female	Hepatitis B	TACE	None	TUR‐Bt	Posterior	Cx; LVB	N/R
6	Miyajima et al. [11]	2023	Japan	83	Male	N/R	Hepatectomy, Cx; ATZ/BEV and LVB	Lung lymph node	TUR‐Bt	Posterior	Cx; LVB	N/R
7	Lorger et al. [12]	2024	Australia	66	Male	Hepatitis C alcoholic	Microwave ablation, TACE, Cx; ATZ/BEV, Rx	Bone adrenal gland	TUR‐Bt	Right anterior	Cx; ATZ/BEV	N/R
8	Our case	2025	Japan	81	Male	Hepatitis C	Hepatectomy, TACE, Cx; ATZ/BEV, Rx	Bone lung	TUR‐Bt	Posterior	BSC	Died of disease 25 days after TUR‐Bt

Abbreviations: ATZ/BEV, Atezolizumab and Bevacizumab; BSC, best supportive care; Cx, chemotherapy; HCC, hepatocellular carcinoma; LVB, Lenvatinib; N/R, not reported; Rx, radiotherapy; TACE, transcatheter arterial chemoembolization; TUR‐Bt, transurethral resection of bladder tumor.

## 4 Conclusions

This report presents a rare case of urinary bladder metastasis from HCC. Although uncommon, secondary lesions should be considered in the differential diagnosis of bladder tumors in patients with a history of HCC.

## Consent

Informed consent was obtained from the subject.

## Conflicts of Interest

The authors declare no conflicts of interest.

## Data Availability

The data that support the findings of this study are available on request from the corresponding author. The data are not publicly available due to privacy or ethical restrictions.
